# Surgical resection of primary tumor improves survival of pancreatic neuroendocrine tumor with liver metastases

**DOI:** 10.18632/oncotarget.19523

**Published:** 2017-07-24

**Authors:** Lianyuan Tao, Dianrong Xiu, Abuduhaibaier Sadula, Chen Ye, Qing Chen, Hanyan Wang, Zhipeng Zhang, Lingfu Zhang, Ming Tao, Chunhui Yuan

**Affiliations:** ^1^ Department of General Surgery, Peking University Third Hospital, Beijing 100191, China

**Keywords:** pancreatic neuroendocrine tumor, liver metastasis, prognosis, surgical resection, survival

## Abstract

This study investigates survival of patients diagnosed with pancreatic neuroendocrine tumor with liver metastases based on local treatment on the primary tumor. Patients diagnosed with stage IV PNET between 2010 and 2014 were identified from the Surveillance Epidemiology and End Results database. Cancer-Specific Survival and Overall Survival were examined. A total of 191 patients with pancreatic neuroendocrine tumor with liver metastases were included in this analysis. There were 47 patients (24.6%) who received surgical resection and 144 (75.4%) who did not. Patients with N1 stage was more likely to be treated with surgical resection. The results showed that surgical resection of primary tumor was associated with Cancer-Specific Survival (*p* = 0.028) and Overall Survival (*p* = 0.025) benefit. Not receiving surgery, being unmarried and N1 stage are factors associated with poor survival. This study reveals that local treatment on the primary benefits both Cancer-Specific Survival and Overall Survival in PNET patients with LM. This may be suggestive for the management on this patient population.

## INTRODUCTION

Pancreatic neuroendocrine tumors (PNETs), originating from cells of the neuroendocrine system, are uncommon and contribute 1.3% to 10.0% of all pancreatic tumors, with an incidence rising at an annual rate of 3.65/10000 people per year [1–3]. PNETs represent a heterogeneous group of neoplasms showing a large variation in tumor behaviors and a wide spectrum of clinical manifestations [3–7]. Furthermore, a set number of PNETs are non-functional with vague clinical symptoms, which led to a significant clinical challenge to diagnosis in clinic work, and many of them were diagnosed when developed distant metastases [7]. By searching the data from the surveillance epidemiology and end results (SEER) database from 2010 to 2014, we found about 21.2% (297/1399) were diagnosed at stage IV, with metastases frequently found in the liver (87.9%, 267/297 of cases). Moreover, liver metastases is the most powerful predictor of survival in PNETs, the 5-year survival rate (75–99%) was significant worse than patients without liver metastasis (13–54%) [1, 8, 9]. Therefore, liver metastasis play a significant role in PNETs. A special analysis on the treatment of PNETs with liver metastasis (PNETLM) is of great significant.

Many study advocate the resection of the primary pancreatic tumor in the setting of metastatic disease. The most recent European Neuroendocrine Tumor Society (ENETS) and North American Neuroendocrine Tumor Society (NANETS) guidelines also recommend removing the primary pancreatic tumor [1, 10, 11]. However, whether such strategy suitable for PNETLM is still lack of clinic evidence. Therefore, we used the surveillance epidemiology and end results (SEER) database to investigate the survival outcomes of patients with PNETLM treated with or without surgical resection of the primary tumor in a contemporary cohort.

## RESULTS

### Patient characteristics

A total of 191 PNET patients with LM were included in the current analysis (Table 1). The median age was 59 years. Most patients were White (*N* = 155, 81.2%) and male patients comprised 61.8% (*N* = 118). There were 47 patients (24.6%) who received surgical resection and 144 (75.4%) who did not. Among the 47 patients who received surgical resection, 25 patients received partial pancreatectomy, 13 patients had local or partial pancreatectomy and duodenectomy, 3 had total pancreatectomy, 2 had total pancreatectomy and subtotal gastrectomy or duodenectomy, and 2 received extended pancreatoduodenectomy. The remained 3 patients’ method of operation is unknown. More than a half of patients were married (*N* = 111, 58.1%). Compared with patients did not received surgical resection of primary tumor, patients in the surgery group was younger (median age: 55 vs 62, *p* = 0.004). There was no significant difference among distributions of gender and race between the groups. The detailed patient characteristics are shown in Table 1.

**Table 1:** Baseline characteristics of metastatic pancreatic patients included in the analysis (*N* = 191)

Characteristics		Total (*N* = 191)	No-surgical resection (*N* = 144)	Surgical resection (*N* = 47)	*P*
**Age (years)**	< 65	116	79	37	0.004
	≥ 65	75	65	10	
**Gender**	male	118	91	27	0.481
	female	73	53	20	
**Race**	white	155	113	42	0.097
	Other	36	31	5	
**Marital status**	Married	111	82	29	0.795
	Other	69	53	16	
	Unknown	11	9	2	
**Tumor location**	Head	53	41	12	0.155
	Body and Tail	84	58	26	
	Overlapping	19	14	5	
	Other	35	31	4	
**Histological grade**	Well	57	35	22	< 0.001
	Moderate	30	15	15	
	Poor	10	5	5	
	Unknown	94	89	5	
**T stage**	T0–T2	67	55	12	< 0.001
	T3–T4	76	42	34	
	Tx	48	47	1	
**N stage**	N0	103	86	17	< 0.001
	N1	58	28	30	
	Nx	30	30	0	

### Factors associated with receipt of surgical resection

To better understand the patient selection, we analyzed the clinicopathological factors associated with removal of primary tumor. As shown in Table 2, the univariate analysis demonstrated that patients with age < 65, T3–T4 and N1 stage were associated with increased possibility to receive surgery. The multivariate analysis showed that patients in N1 stage were more likely to be treated with surgical resection.

**Table 2:** Factors associated with receipt of surgical resection of the primary tumor

Variables			Univariate model		Multivariate model		
		OR	(95% CI)	*P*	OR	(95% CI)	*P*
**Gender**	Male	1(Referent)			1(Referent)		
	Female	1.272	0.651–2.486	0.482	1.756	0.627–4.918	0.284
**Age (years)**	< 65	1(Referent)			1(Referent)		
	≥ 65	0.328	0.152–0.711	0.005	0.602	0.200–1.817	0.368
**Race**	White	1(Referent)			1(Referent)		
	Other	0.434	0.158–1.19	0.105	0.305	0.065–1.441	0.134
**Marital status**	Married	1(Referent)			1(Referent)		
	Other	0.854	0.423–1.721	0.658	0.704	0.234–2.115	0.532
	Unknown	0.628	0.128–3.08	0.567	0.577	0.062–5.405	0.63
**Tumor location**	Head	1(Referent)			1(Referent)		
	Body and Tail	1.532	0.693–3.383	0.292	1.763	0.550–5.650	0.34
	Overlapping	1.22	0.365–4.079	0.747	1.049	0.168–6.563	0.959
	Other	0.441	0.13–1.499	0.19	1.028	0.188–5.615	0.974
**Histological grade**	Well	1(Referent)			1(Referent)		
	Moderate	1.591	0.652–3.884	0.308	1.08	0.308–3.785	0.905
	Poor	1.591	0.413–6.133	0.5	1.158	0.214–6.267	0.865
	Unknown	0.089	0.031–0.255	< 0.001	0.069	0.019-.255	< 0.001
**T stage**	T0–T2	1(Referent)			1(Referent)		
	T3–T4	3.71	1.716–8.021	0.001	1.891	0.648–5.518	0.244
	Tx	0.098	0.012–0.778	0.028	0.08	0.007–0.926	0.043
**N stage**	N0	1(Referent)			1(Referent)		
	N1	5.42	2.607–11.27	< 0.001	7.428	2.529–21.820	< 0.001
	Nx	None			None		

### Survival outcomes

**Figure 1:**
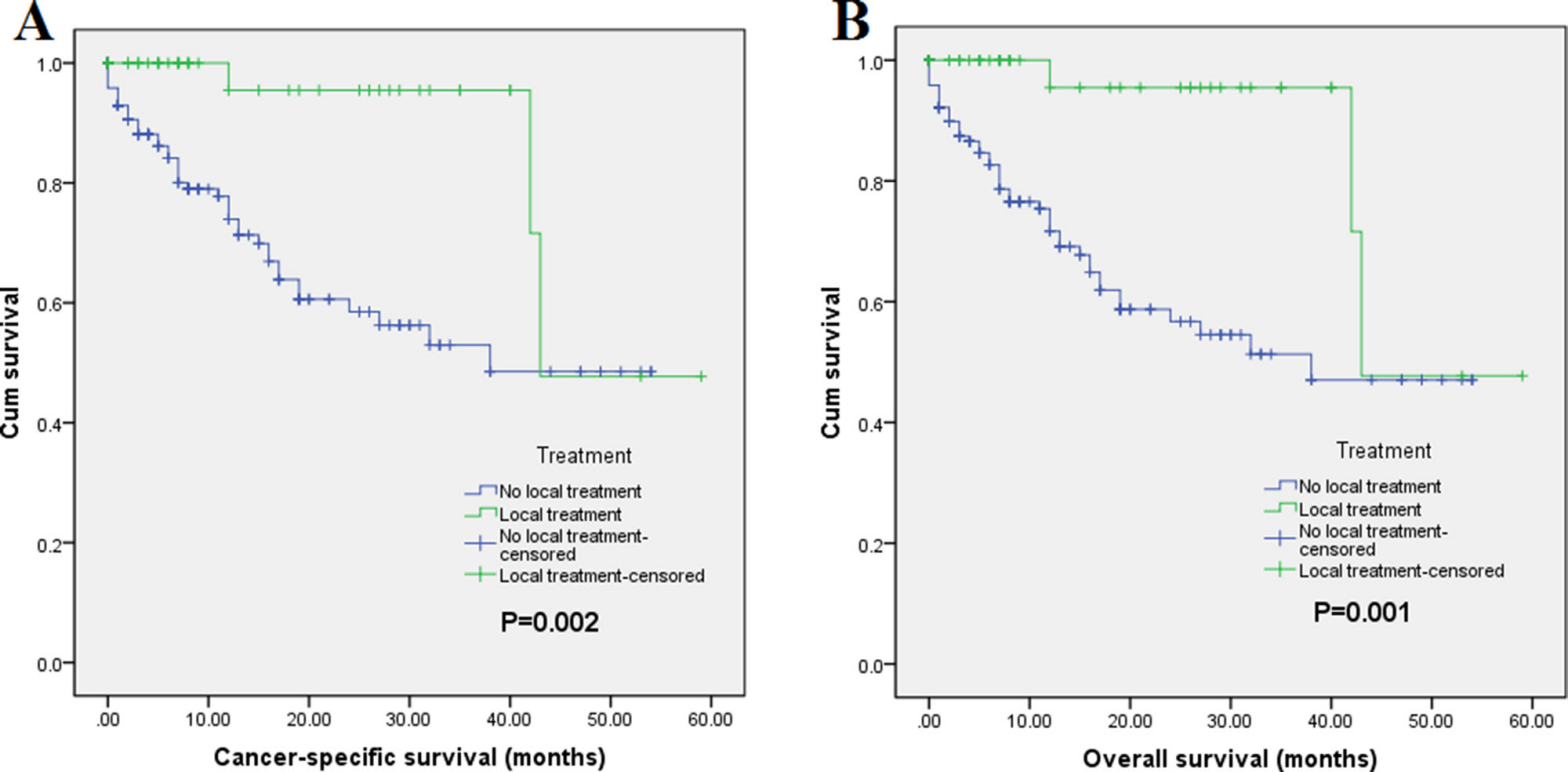
Survival curves with log-rank test of (**A**) CSS (***p*** < 0.001) and (**B**) OS (***p*** < 0.001).

Of a total 191 patients, mortality occurred in 48 (25.1% of 191) patients at the end of follow-up. And 45 (23.6% of 191) patients were dead due to PNET. Regarding Cancer-Specific Survival (CSS), the 1-year CSS rates were 95.5% in surgery group and 74% in non-surgery group, and the 3-year Overall Survival (OS) rates were 95.5% and 48.5% in surgery group and non-surgery group, respectively. The median survival time were 49.0 months (95% CI = 40.6–57.4) for surgery group and 35.6 months (95% CI = 28.8–38.4) for non-surgery group (*p* < 0.001). Concerning OS, the 1-year OS rates were 95.5% and 71.7% in surgery group and non-surgery group, and their 3-year OS rates were 95.5% and 47%, respectively. The median survival time were 49.0 months (95% CI = 40.6–57.4) for surgery group and 32.7 months (95% CI = 27.9–37.4) for non-surgery group (*p* < 0.001). The survival curves of CSS and OS are shown in Figure 1. Multivariate Cox regression analysis revealed that receipt of surgical resection was associated with better CSS (HR = 0.197, 95% CI = 0.046–0.833) and OS (HR = 0.199, 95% CI = 0.048–0.819) (Table 3). Moreover, the results also demonstrated that being unmarried and advanced N stage were associated with poor CSS. In addition, poor OS was also inclined to be occurred in patients with being unmarried and N1 stage. Taken together, these data suggest the high risk population of patients with PNETLM.

**Table 3:** Multivariate analysis of cancer-specific survival (CSS) and overall survival (OS) in metastatic pancreatic cancer

Variables		Overall survival			Cancer-specific survival		
		HR	95.0% CI	*P*	HR	95.0% CI	*P*
**Gender**	Male	1(Referent)			1(Referent)		
	Female	1.186	0.614–2.292	0.611	1.113	0.559–2.215	0.761
**Age (years)**	< 65	1(Referent)			1(Referent)		
	≥ 65	1.743	0.909–3.344	0.094	1.763	0.897–3.463	0.1
**Race**	White	1(Referent)			1(Referent)		
	Other	0.667	0.293–1.522	0.336	0.707	0.304–1.64	0.419
**Treatment**	No surgical resection	1(Referent)			1(Referent)		
	Surgical resection	0.199	0.048–0.819	0.025	0.197	0.046–0.833	0.027
**Marital status**	Married	1(Referent)			1(Referent)		
	Other	2.349	1.222–4.518	0.01	2.482	1.264–4.874	0.008
	Unknown	0.541	0.069–4.246	0.559	0.563	0.071–4.443	0.586
**Tumor location**	Head	1(Referent)			1(Referent)		
	Body and Tail	1.251	0.562–2.783	0.583	1.306	0.565–3.018	0.532
	Overlapping	0.903	0.276–2.949	0.866	1.011	0.305–3.356	0.986
	Other	2.052	0.877–4.803	0.098	2.076	0.859–5.019	0.105
**Histological grade**	Well	1(Referent)			1(Referent)		
	Moderate	0.789	0.152–4.105	0.778	0.789	0.151–4.135	0.779
	Poor	3.093	0.656–14.578	0.153	3.198	0.664–15.405	0.147
	Unknown	3.04	1.214–7.61	0.018	2.831	1.119–7.162	0.028
**T stage**	T0–T2	1(Referent)			1(Referent)		
	T3–T4	0.892	0.423–1.878	0.763	0.849	0.393–1.834	0.676
	Tx	0.806	0.317–2.047	0.65	0.74	0.279–1.963	0.545
**N stage**	N0	1(Referent)			1(Referent)		
	N1	2.889	1.283–6.509	0.01	3.361	1.449–7.795	0.005
	Nx	1.326	0.52–3.378	0.555	1.674	0.636–4.404	0.297

## DISCUSSION

Liver metastases have been highly observed in PNET when diagnosed, and which are also one of the most significant prognostic factor. Although surgical resection has been suggested as the mainstay treatment by American Society of Clinical Oncology Clinical Practice Guideline, few studies investigate the role of surgical resection in treatment of PNETLM. Keutgen, et al. used the SEER database to show that resection of the site of the primary nonfunctioning PNET is associated with greater survival in patients with distant metastases and could therefore be considered as a additional treatment option [12]. However, such study have not distinguish liver metastasis from other site metastasis, which play a specialized and key role in PNETs.

We explored the association between local treatments on PNETLM and the survival outcomes relying on SEER database. In order to make clear distinctions between liver metastasis and other metastasis and give a certain analysis on the liver metastasis in PNETs, we excluded the patients with other metastasis, have other primary tumors and those lost the surgical information on lymphatic metastasis. Analysis of factors associated with receipt of surgical resection indicate that atients in N1 stage is likely to be treated with surgery. This result may because patients who received primary tumor surgical resection may have more chance to find lymph node metastasis during operation than those did not who may mostly evaluated the lymph node status based on imaging examination. Further analysis showed being married was associated with survival benefit. Married status is shown to play a favorable prognostic role in various cancers [13–15], which may owe to the potentially significant impact of social support on cancer treatment and survival. In addition, multivariate Cox regression confirmed that patients receiving surgical resection of primary tumor had better CSS and OS.

It has been demonstrated that a lower tumor burden at baseline is associated with better prognosis [16–19], which may because debulking surgery may improve the effect of subsequent local treatment for metastases. For example, debulking surgery previous to PRRT could have a radiobiological rationale, since the morphology of smaller lesions usually allows higher dose concentrations, resulting in a higher chance of tumor response [16]. On the other hand, the resection of the primary tumor in the setting of liver metastasis has been showed to benefit overall survival (OS) [17]. It has been proved that primary tumor resection prior to PRRT can be safely proposed in G1–G2 PNETs with diffuse liver metastases because it seems to enhance response to PRRT and to improve prognosis [17].

The biological mechanisms of liver metastasis in PNETs has seldom reported for its low incidence. However, such progression may similar to pancreatic cancer. Firstly, liver recognized as the most common site of pancreatic tumor metastasis most due to anatomical situation [20]. Secondly, Circulating tumor cells (CTCs) can also colonize their tumors of origin, which is termed “tumor self-seeding” [21]. A subpopulation of migrating cancer stem cells (CSC) is also essential for such metastasis [22]. Thirdly, “metastatic niche” may already existed in the liver even before the metastases formed [23, 24], a tumor microenvironment may created with help of a variety of immune cells [23, 25] or cytokines [26]. These changes in tumor biology during metastasis in pancreatic cancer may be in common with PNET, which also experienced a high ratio of liver metastasis. Such change could be implicated in the process of tumor cells dissemination and also shed light on the rationale for primary tumor resection.

There are several limitations to the present study. First, it is limited by the retrospective nature; therefore, selection bias could occur. Second, demographic information provided by the SEER database did not include functional classification, comorbidity, performance status, smoking, alcohol consumption and other detailed factors. The contribution of these factors to the survival benefit could not be evaluated. And these confounders could not be adjusted in multivariate analysis. Third, data on therapy for liver metastasis are not included in SEER database. As therapy for liver metastasis is the mainstay treatment for PNETLM, the impact of surgery on systemic therapy could not be estimated. Last, the SEER database provide the data about the location of distant metastasis since 2010, and the most recently data about PNET was 2014, therefore, only patients from 2010 to 2014 were involved. However, the sample involved in this study is enough, as a positive association was found between primary tumor resection and long survive.

Despite the stated limitations, our study reveals that local treatment on the primary benefits both CSS and OS in patients with PNETLM. This may be suggestive for the management on this patient population. Further prospective trails are still needed to validate our results.

## MATERIALS AND METHODS

### Patient cohort

**Figure 2:**
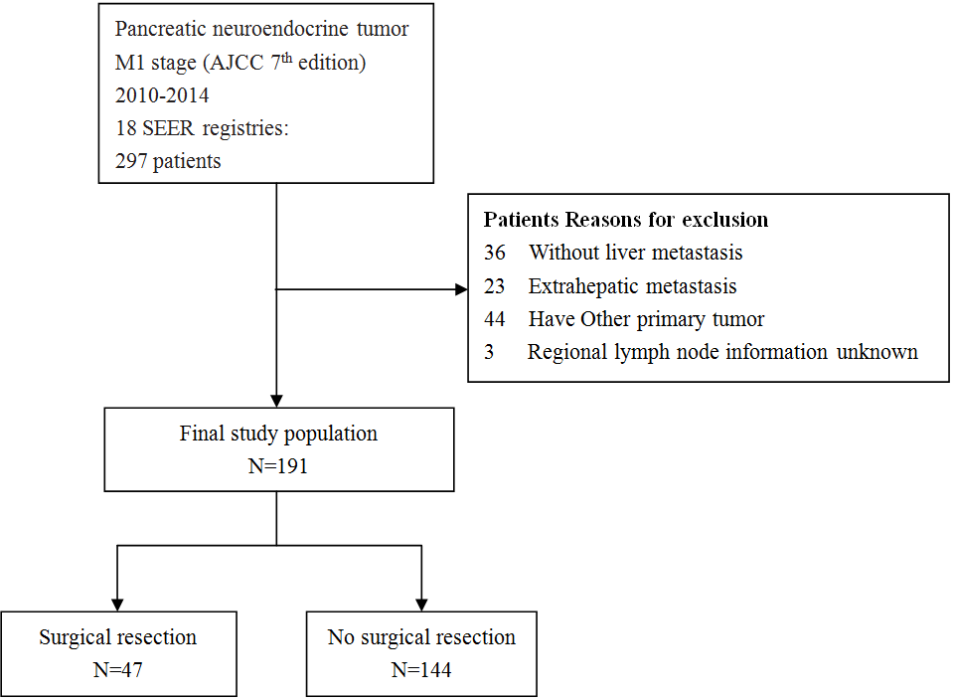
Flowchart of selection process of eligible patients from SEER database

The data of this study were extracted from SEER-18 registry of the National Cancer Institute. The database is publicly available and we retrieved the data using SEER*Stat Software Version 8.3.4. Because the SEER database used deidentified data, this study was exempted from institutional review board oversight. We identified patients diagnosed between January 1, 2010 and December 31, 2014 with a primary site of ‘pancreas’, with American Joint Committee on cancer (AJCC) stage (7th edition) IV and with International Classification of Diseases for Oncology, Third Edition (ICD-O-3) codes 8150/3, 8151/3, 8152/3, 8153/3, 8155/3, 8156/3, 8240/2, 8240/3, 8241/3, 8242/3, 8243/3, 8246/2 and 8249/3 from SEER database. Patients without liver metastases, with extrahepatic metastases, with other primary tumor, unknown regional lymph node information (scope regional lymph node surgery) were excluded. The process of patient selection is shown in Figure 2.

#### Data collection

The following demographic information of each patient was collected: age at diagnosis, gender, primary site of tumor, T stage, N stage, M stage, surgical resection of the primary site (yes or no), marital status, SEER cause-specific death classification, survival months, and vital status. Pancreatic cancer-specific survival (CSS) was defined as the time from diagnosis to death from pancreatic cancer and OS was defined as the duration from diagnosis to death from any cause. Information on systemic treatment was not provided by SEER database.

#### Statistical analysis

The primary endpoint of this study was CSS and the secondary endpoint was OS. Chi-square test was utilized to compare the differences in clinical and demographic features between patients treated with or without surgical resection. CSS and OS were examined by using the Kaplan-Meier method with log-rank test. The associations between demographic factors with receipt of surgical resection were evaluated using Logistic regression analysis. Multivariable survival analyses of CSS and OS were conducted using the Cox proportional hazards model. *P* < 0.05 was considered as statistically significant. All statistical analyses were performed using IBM SPSS Statistics 22.0 (IBM, Armonk, NY, USA).

### Institutional review board statement

This study was approved by the Clinical Ethics Committee of Peking University Third Hospital.
